# Impact of parathyroid gland classification on hypoparathyroidism following total thyroidectomy with central neck dissection for differentiated thyroid cancer

**DOI:** 10.1080/07853890.2025.2525392

**Published:** 2025-06-26

**Authors:** Mohammad Aitzaz Hassan, Ambreen Javed, Muhammad Abdullah Ali

**Affiliations:** a Gomal Medical College, Dera Ismail Khan, Pakistan; b Khyber Medical College, Peshawar, Pakistan

We were intrigued by the article by Sheng et al. [1] discussing the effects of parathyroid gland (PG) classification on the incidence of postoperative hypoparathyroidism post total thyroidectomy with central neck dissection. The suggested four-type classification of PGs is relevant from a clinical perspective and also highlights the important step of intraoperative preservation of PGs as a practical surgical concern.

We would, however, like to make some comments that, in our opinion, could enhance the clinical relevance of their findings and improve the methodological rigor of the study. To begin with, the surgical team involved in the PG classification also performed the surgeries, meaning the study utilized an unblinded design. This poses a risk of observer bias because understanding the positioning of the glands and their likely outcomes can shape the postulated classification [2]. Also, the identification of parathyroid glands (PGs) relied purely on visual observation. This method is too subjective and can lead to misidentification of glands during surgery. For example, indocyanine green fluorescence angiography and near-infrared autofluorescence are more current intraoperative techniques that can better identify and preserve PGs [3]. Although the authors indicated that all patients had their parathyroid hormone (PTH) levels normalized by six months, there was no mention of calcium levels following the same longitudinal trend analysis. Delayed or persistent hypocalcemia symptoms can be experienced by a patient even after normalization of PTH levels. Clinical recovery from hypoparathyroidism mostly rests on normalization of calcium and resolution of symptoms besides biochemical PTH normalization. Ignoring monitoring and reporting these might lead to undetected cases of normo-PTH hypocalcaemia or such things as prolonged recovery [4]. Last but not the least, further studies should incorporate blinded assessors, employ more than one PG localization method, and follow standard guidelines such as CONSORT for surgical trials into the future for better reproducibility and transparency [4].

In conclusion, Sheng and colleagues have provided insight into parathyroid preservation through a novel classification scheme; however, their investigation stands to gain methodological refinements that would improve clinical relevance and generalizability. We appreciate their work and encourage future endeavours that synergize modern surgical technologies and rigorous outcome tracking.

## Data Availability

This Letter to the Editor is a critique of a previously published article and does not involve the generation or analysis of new data. Therefore, no data are available.
